# The Bidirectional Relationship Between Subjective Well-Being and Academic Achievement in Adolescence

**DOI:** 10.1007/s10964-021-01413-3

**Published:** 2021-03-06

**Authors:** Cristian Bortes, Susanne Ragnarsson, Mattias Strandh, Solveig Petersen

**Affiliations:** 1 Department of Social Work, Umeå University, SE-901 87 Umeå, Sweden; 2 Department of Epidemiology and Global Health, Umeå University, SE-901 87 Umeå, Sweden; 3 Centre for Research on Child and Adolescent Mental Health, Karlstad University, SE-651 88 Karlstad, Sweden

**Keywords:** Academic achievement, Bidirectional associations, Cross-lagged panel analysis, Gender differences, Subjective well-being

## Abstract

The well-being of young people in relation to their school performance has received increased attention in recent years. However, there is a lack of knowledge about the longitudinal and reciprocal relationship between adolescents’ subjective well-being and their academic achievements. The current study examined the bidirectional relationship between subjective well-being and academic achievement across two timepoints (T1 and T2) during the course of mid to late adolescence, i.e., in school year 9 (age 15), and school years 11–12 (ages 17–18). The study also investigated variation in the association as a function of adolescent gender. Data on subjective well-being and teacher-assigned school grades of 723 adolescents (48.7% girls) residing in Sweden were analyzed by estimating a series of cross-lagged path models. The findings suggest gender differences in the relationship as no associations were found among boys. Support for a bidirectional relationship between the constructs was only found for girls. For girls, higher subjective well-being at T1 was associated with higher academic achievements at T2, while higher academic achievements at T1 was associated with lower subjective well-being at T2. These findings highlight that the subjective well-being of adolescent girls may be important for their ability to perform at school, but their academic achievements may also inflict negatively on their subjective well-being.

## Introduction

Despite a growing interest in adolescents’ well-being in relation to their achievements at school, existent literature on the topic has two major limitations. First, most previous studies have focused on how well-being influences student achievement (see Amholt et al., 2020 for a recent review), and thus regarded the relationship as unidirectional. But the educational outcomes that students succeed or fail to achieve are also likely to affect their well-being. However, there is a lack of research on the reciprocity between adolescents’ subjective well-being and their academic achievements. Second, most studies have examined well-being and achievement at a single point in time. Therefore, while prior research has established that a general correlation between subjective well-being and academic achievement may exist (see Bücker et al., 2018 for a meta-analysis), it does not provide insights into changes in this relationship over time. To address these important gaps in the literature, the present study investigated the bidirectional relationship between subjective well-being and school grades across two timepoints during mid to late adolescence.

### The Bidirectional Relationship Between Subjective Well-Being and Academic Achievement

Various theories and measures of subjective well-being have been proposed (Diener, 2009), but it has often been defined as a construct with cognitive and affective dimensions comprising three components: life satisfaction, positive, and negative affect. Life satisfaction, the cognitive dimension of subjective well-being, refers to a person’s overall evaluation of the quality of his or her life. Positive affect refers to a person’s tendency to feel positive, such as happiness, while negative affect refers to the tendency to feel distress. Modifications to measure these dimensions within specific context, such as the school, are common. In such cases, students are asked about their satisfaction with school, enjoyment of school, and the absence of worries at school.

Subjective well-being is frequently thought to play a role in education for reasons based on the broaden-and-build theory of positive emotions (Fredrickson, 2001). According to this theory, positive emotions broaden a person’s mindset and, in educational contexts, increase attention to learning, which in turn builds personal resources and skills. In contrast, negative emotions, such as sadness and anxiety, are thought to narrow a person’s cognitions, thereby hindering learning. These ideas have received empirical support from studies showing that students with higher levels of positive affect tend to be more engaged and those with higher levels of negative affect less engaged in school (see the studies in King et al., 2015). Life satisfaction has also been positively and reciprocally linked to levels of school engagement (Datu & King 2018; Lewis et al., 2011), suggesting that it may be both an antecedent and consequence of a student’s degree of engagement in school (Salmela-Aro & Tuominen-Soini, 2010). School engagement, in turn, is widely acknowledged as a key determinant of successful academic outcomes (Lee, 2014). Thus, there are both theoretical and empirical reasons to expect that subjective well-being may lead to better academic achievements.

However, the relationship is not necessarily simply unidirectional. Exhaustive engagement may be positively related to short-term academic performance, but may also lead to school-related burnout and less engagement in the long-term (Walburg, 2014). School burnout can be defined as a combination of exhaustion due to study demands, cynicism towards school, and feelings of inadequacy as a student (Salmela-Aro et al., 2009). It may be caused by long-term school-related stress and pressure to achieve; more specifically by discrepancies between school workload, students’ internal resources and expectations of school results (Wang et al., 2015). A related concept is “effort–reward imbalance”, which in a school setting means that students are more likely to experience stress-related ill-health when they perceive a lack of reciprocity between their efforts at school and the rewards, e.g., a lower than expected score for a test (Låftman et al., 2014). Although school burnout and subjective well-being are different constructs, there is some overlap and correlation between the two. For example, high burnout has been found to be indicative of subsequent low subjective well-being (Raiziene et al., 2014). Hence, while subjective well-being may promote educational achievements by increasing school engagement, exhaustive engagement in the pursuit of high achievements could take its toll on well-being. Note that students’ school engagement is here discussed as a part of conceptualizing the relationship between subjective well-being and academic achievement, but is not empirically analyzed in the current study.

Two prior studies have explicitly investigated the longitudinal reciprocal relationship between specific achievement measures and a component of subjective well-being. In the first (Ng et al., 2015), positive reciprocal relations were found between overall life satisfaction and school grades of 821 students of one suburban middle school in southwestern USA. The study had a short-term longitudinal design, data were collected in two waves with a 5-month interval, and the participants included 7^th^ and 8^th^ grade students. Their findings indicate, with standard caveats regarding generalization and assumptions that results apply to students in schools with different cultural characteristics, that well-being and academic achievement may be mutually reinforcing. Thus, well-being and high school grades may have synergistic effects, at least within a short time interval (5 months) in early adolescence. In the other study (Steinmayr et al., 2016), the reciprocal relationship between components of subjective well-being and grade point averages (GPAs) of 290 German high school students were examined, based on measurements when they were in 11^th^ and 12^th^ grade. GPA at the first measurement occasion was positively related to changes in life satisfaction between the two occasions, but no component of well-being provided predictive indications of changes in GPA. Thus, their findings do not support the assumption that students are more academically successful the happier they are. In summary, due to the paucity of studies and lack of robust consistent findings, there is substantial need for clarification of the bidirectional relationship between educational achievements and subjective well-being.

### The Role of Adolescent Gender

There are several well-established between-gender differences in adolescents’ educational performance and general well-being. While girls generally tend to get higher grades than boys (Voyer & Voyer, 2014), they also experience higher demands and stress related to school (Giota & Gustafsson, 2017), in many regards due to peers and parents harboring and expressing higher expectations for their academic performance (Östberg et al., 2015). Partly for this reason, high performing girls tend to report poor well-being despite relatively high achievements (Låftman et al., 2013). Concerning boys, they too develop high burnout levels in secondary school, though girls’ levels are higher (Salmela-Aro & Tynkkynen, 2012). However, these associations are based on health complaints, stress-related symptoms and other “deficit”-oriented measures of well-being (or rather ill-being). In contrast, subjective well-being is rooted in the paradigm of positive psychology (Seligman & Csikszentmihalyi, 2000), and focuses on “strength”-oriented measures of well-being (positive health). To date, there is only one previous investigation of any component of subjective well-being, its association with a student achievement measure, and the potential moderating role of gender. That study found that boys and girls with lower than average levels of life satisfaction obtained similar GPAs, but as life satisfaction increased girls tended to obtain higher GPAs and math test scores than boys (Heffner & Antaramian, 2016). The study design was cross-sectional, the participating students attended the same middle school in the USA, and they were all in 7^th^ and 8^th^ grades. Moreover, the emphasis was still on “well-being-to-achievement effects”. Thus, questions still remain regarding the influence of gender in the bidirectional relationship between subjective well-being and academic achievement, especially among older adolescents and in other contexts.

### The Transition from Compulsory to Upper Secondary Education

In Sweden, children start compulsory school in the year in which they turn 6 years old (pre-school class) and finish after 10 years at the age of 15–16 years (school-year 9). The transition from compulsory education to a vocationally or academically oriented program in upper secondary school is a major educational change during adolescence. This transition entails increased academic pressure and individual responsibility, as well as increased social demands related to manage new peer groups. With regard to academic achievement, prior achievement tends to be a strong predictor of future achievement (Khattab, 2015). Therefore, youth who performed well by the end of compulsory education can be expected to also perform well in upper secondary school. While achievement is usually stable across time, well-being generally declines from early through mid and late adolescence, especially among girls (Herke et al., 2019). To date, however, no prior study has investigated the longitudinal relationship between subjective well-being and academic achievement within the context of this transition in Sweden.

## Current Study

This study responds to calls for more longitudinal research of the reciprocal relationship between subjective well-being and academic achievement. The main objective was to explore the directionality of the association between subjective well-being and academic achievement. Three specific research questions were addressed. First, can changes in adolescents’ subjective well-being between two timepoints be predicted from their academic achievements at the first timepoint, and does their subjective well-being at one timepoint influence subsequent changes in their achievements? Second, which of these cross-lagged associations is stronger? Third, how do these associations vary between boys and girls?

## Methods

### Participants

Data for this study were obtained from the longitudinal “Study of Health in School-Children in Umeå” (SISU). Umeå is a university municipality with ~120,000 inhabitants in northern Sweden. The current study addressed all 974 adolescents in school-year 9 in 2009. The data consisted of responses to a questionnaire that adolescents completed, during regular school hours, in school-year 9 of compulsory school, when they were ~15 years old (T1), and 2 years later, in school-year 2 of upper secondary school (end of school-year 11), when they were ~17 years old (T2), provided that they were still living in Sweden. The adolescents’ parents also completed a short questionnaire at home (for a more detailed description of SISU see Petersen et al., 2009). In addition to the questionnaire, teacher-assigned school grades from the end of compulsory school (school-year 9) and the end of upper secondary school (school-year 12) were obtained from school registers. For simplicity, the second measure of academic achievement (grade 12) and the second measure of subjective well-being (end of school year 11) are jointly labeled as T2 measures. Of the target population, 939 adolescents participated at T1, but only 893 provided complete data at T1 on both well-being and school grades (internal missing: well-being *n* = 4, grades *n* = 42). Of these 893 adolescents, 807 agreed to participate also at T2, but three of those did not provide complete data at T2 on well-being, and grades were lacking in another 81. The main reason for the lack of school grades in these students was difficulties identifying the records, for instance due to students moving abroad during the last year of upper secondary school or attending school forms that did not archive grades over time. Taken together, the analytic sample for this study included 723 adolescents (48.7% girls), which corresponds to a participation rate of 74.2%. Most of the participants were born in Sweden (93.9%) and had at least one parent who had been born in Europe or North America (88.4%). At T1, most of the participants lived in a household with both parents (68.2%), and had at least one parent who had completed a college or university education (71.8%).

### Attrition Analysis

A series of chi-square tests were conducted to examine whether the adolescents who did not participate in the study (0 = missing) differed from the participating adolescents (1 = no missing) in any systematic way based on their sociodemographic characteristics (gender, immigrant background, family structure). The results indicated a statistically significant difference between the two groups with regard to family structure (*p* = 0.001), suggesting that adolescents with a family structure other than living with both parents (e.g., single parent) were more likely to drop out. However, no other statistically significant difference between the two groups were found neither in terms of gender (*p* = 0.141) nor immigrant background (*p* = 0.238). Thus, based upon these results there seems to be no missing bias, except with regard to family structure.

### Measures

#### Subjective well-being

Subjective well-being was measured using a scale from the KIDSCREEN-52 instrument (The KIDSCREEN Group Europe, 2006). The instrument was designed to assess the health-related quality of life of children and adolescents aged 8–18 years and has previously demonstrated acceptable reliability and validity (Ravens-Sieberer et al., 2014). For this study, the scale that reflects positive emotions and satisfaction with life through six questions (items) was used: “Thinking about the last 4 weeks… Has your life been enjoyable?; Have you felt pleased that you are alive?; Felt satisfied with your life?; Been in a good mood?; Felt cheerful?; Had fun?”. These questions were scored on a five-point response scale ranging from “not at all/never” to “always”. Following the KIDSCREEN handbook (The KIDSCREEN Group Europe, 2006), answers were (re)coded so that higher values indicate better well-being. A sum score was generated, which was first transformed into Rasch person parameters, then further into *z*-values and finally into *t*-scores with scale means around 50 and standard deviations around 10. Adolescents with more than one question unanswered were counted as missing. Note that this provides a global measure of subjective rather than context-specific well-being. It also only covers the positive (excluding negative) perceptions and emotions self-reportedly experienced by the respondent, and their satisfaction with life as a whole. Thus, it is intended to capture two of the three components of subjective well-being. A low score indicates little pleasure in life whilst a high score indicates that the respondent is happy and satisfied with life. For brevity, this measure and the construct subjective well-being is referred to as “well-being” hereafter.

#### Academic achievement

Academic achievement was measured using the school grades assigned to students by teachers in accordance with the Swedish grading system at T1 and T2. Academic achievement at T1 comprised the sum of the 16 highest subject grades obtained by the student in her/his final year of compulsory schooling (9^th^ grade). For each subject, a student could obtain a grade ranging from 0 (indicating that the minimal knowledge requirements for that subject were not achieved) to 20, so the possible sum ranged from 0 to 320 and indicated the general academic achievement at that timepoint. Academic achievement at T2 were grade point averages obtained in the final year of upper secondary schooling (12^th^ grade). In upper secondary school, students take different courses, with varying credits. For each course, a student could obtain a grade ranging from 0 to 20, as in the compulsory school system, but in the upper secondary school grading system this grade is multiplied by the credits for the particular course. Thus, the final grade obtained when graduating was the sum of each grade divided by the total educational credits (at least 2500 credits), thus yielding a grade point average. In order to enable comparison between the two time points, the two measures of academic achievement were standardized by converting them to *z*-scores (mean = 0, standard deviation = 1).

#### Sociodemographics

A short questionnaire completed by the parents was used to capture parental level of education (0 = no parent with a college/university degree, 1 = at least one parent with a college/university degree). The participating students answered questions about sex (0 = boy, 1= girl), family structure (0 = living with both parents, 1 = other, e.g., with a single parent), and immigrant background (0 = both parents born in Europe/North America, 1 = at least one parent born outside Europe/North America). The categorizations of these variables have previously been shown to discriminate for health issues (Petersen et al., 2009). These sociodemographic variables were included as control variables in all the models.

### Data Analysis

Independent sample *t*-tests were used to examine mean differences in well-being and academic achievement between boys and girls. The direction of associations between well-being and academic achievement was examined by comparing alternative cross-lagged path models, by following a model comparison approach (Kline, 2016), conducted in four steps, using Stata v. 16. First, in the baseline model (A), the autoregressive paths were specified, which describe the stability of individual differences in the measured construct from one timepoint to the next. A small (closer to zero) autoregressive coefficient indicates less stability in the construct from the previous timepoint, while a larger (closer to 1) autoregressive coefficient indicates more stability from the previous timepoint (Selig & Little, 2012). In the baseline model we also specified the cross-sectional correlations between the constructs at each timepoint. Then, in the first alternative model (B), a directional path from well-being to academic achievement was added. In the second alternative model (C), a directional path from academic achievement to well-being was added. Finally, in the fourth model, the bidirectional model (D), paths from both well-being and achievement were included simultaneously. In addition, multiple group analyses were performed, with gender as a grouping variable, comparing results for boys and girls.

The models were assessed by combined use of several model fit statistics and information criteria: The Comparative Fit Index (CFI) and Tucker-Lewis Index (TLI), which should both be close to or exceed 0.95 (Hu & Bentler, 1999), and the Root Mean Square Error of Approximation (RMSEA), which should be close to or below 0.06. The Akaike Information Criterion (AIC) and the Bayesian Information Criterion (BIC) were also used to evaluate the relative goodness of fit. For each criterion, the model with the lowest value relative to the other models should be favored (Kuha, 2004). Differences in fit between nested models were tested using chi-square difference tests. Parameters in all models were estimated using the Full Information Maximum Likelihood (FIML) method, which accounts for missing data. This method identifies parameter values with the highest probability of producing the sample data based on all available data, complete or incomplete (Enders, 2010). All models were adjusted for the sociodemographic variables (parental level of education, family structure, and immigrant background). All estimates presented are standardized. The significance level was set at 95%.

## Results

Descriptive statistics for the measures of well-being and academic achievement are presented in Table 1, showing difference in means of both grades and well-being between boys and girls at T1 and T2. At both timepoints, boys reported higher levels of well-being but obtained lower grade points, on average, than girls. Correlations among all variables used in the analysis are presented in Table 2. Well-being at T1, but not at T2, was positively correlated with achievement at both T1 and T2 among girls. Among boys, neither of these variables were correlated.

**Table 1:** Descriptive statistics for the main study variables by gender

	Boys (*n* = 371)				Girls (*n* = 352)				Boys–Girls *t*-test	
	Min	Max	Mean	SD	Min	Max	Mean	SD	Mean diff.	*t*
Academic achievement										
T1	−3.35	1.64	0.01	0.77	−3.20	1.64	0.37	0.84	−0.37^***^	−6.17
T2	−3.25	1.63	−0.12	0.92	−3.25	1.63	0.26	0.94	−0.37^***^	−5.43
Subjective well-being										
T1	9.86	68.49	51.16	10.72	25.23	68.49	45.97	8.67	5.20^***^	7.16
T2	9.86	68.49	50.72	10.67	20.36	172.30	46.70	10.69	4.02^***^	5.06

***p < 0.001

**Table 2:** Correlations of the study variables by gender

	1	2	3	4	5	6	7
1. Immigrant background^a^	–	0.12^*^	−0.11^*^	−0.01	0.10	−0.15^**^	−0.22^***^
2. Family structure^b^	0.06	–	−0.03	−0.03	0.05	−0.25^***^	−0.21^***^
3. Parental education^c^	0.00	0.05	–	0.09	−0.07	0.32^***^	0.28^***^
4. Subjective well-being T1	0.06	−0.00	−0.02	–	0.32^***^	0.19^***^	0.24^***^
5. Subjective well-being T2	0.07	0.02	0.03	0.44^***^	–	−0.06	−0.02
6. Academic achievement T1	−0.05	−0.03	0.31^***^	0.02	−0.05	–	0.74^***^
7. Academic achievement T2	−0.11^*^	−0.03	0.23^***^	−0.03	−0.02	0.72^***^	–

Coefficients below diagonal are for boys, above diagonal are girls

*p < 0.05, **p < 0.01, ***p < 0.001

^a^Immigrant background was coded 0 = both parents born in Europe/North America, 1 = at least one parent born outside Europe/North America

^b^Family structure was coded as 0 = living with both parents, 1 = other (e.g., with single parent)

^c^Parental education was defined as 0 = no parent with a college/university degree, 1 = at least one parent with a college/university degree

Table 3 shows the goodness-of-fit statistics for the four models that were compared for boys and girls. The results from the chi-square difference test (farthest to the right in the table) showed that there were statistically significant differences in fit among all the models. Clearly, the bidirectional model (Model D), which includes paths from both well-being and achievement simultaneously, provides the best fit in terms of all the statistics. This supports the notion that adolescent well-being and academic achievement may have reciprocal effects over time.

**Table 3:** Goodness-of-fit statistics for the tested models

	Goodness-of-fit statistics							χ^2^ difference test	
	RMSEA	CFI	TLI	AIC	BIC	χ^2^ (df)	*p*	vs. model	*p*
Model A^a^	0.064	0.969	0.931	15812	16059	39.89 (16)	0.001	–	–
Model B^b^	0.056	0.979	0.947	15806	16063	30.02 (14)	0.044	A	0.007
Model C^c^	0.061	0.976	0.937	15809	16066	32.96 (14)	0.003	A	0.031
**Model D**^**d**^	**0.050**	**0.986**	**0.958**	**15803**	**16069**	**22.98 (12)**	**0.028**	A/B/C	0.002/0.030/0.007

All models include the socio-demographic control variables at T1 and cross sectional correlated error terms between subjective well-being and academic achievement. Preferred model in bold

^a^Only auto-regressive paths and cross-sectional correlations

^b^Subjective well-being at T1 predicts academic achievement at T2

^c^Academic achievement at T1 predicts subjective well-being at T2

^d^Bidirectional effects of subjective well-being and academic achievement over time

**Fig. 1:**
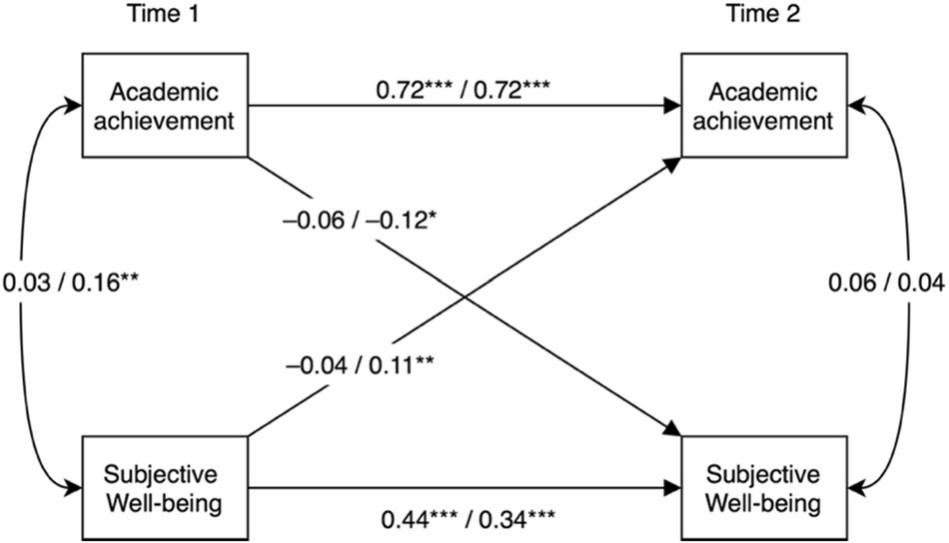
Estimated cross-lagged path model relating academic achievement and subjective well-being (boys *n* = 371, girls *n* = 352). Estimates (standardized) are displayed as “boys”/“girls”. For clarity, the control variables (immigrant background, family structure, parental education) and error terms have been omitted from the figure. *p < 0.05, **p < 0.01, ***p < 0.001

Regarding the autoregressive associations, as can be seen in Fig. 1, the path coefficient for academic achievement from T1 to T2 is close to one, for both boys (0.72) and girls (0.72). This indicates that the adolescents’ standings on this construct were very stable across the two timepoints. In contrast, the autoregressive path coefficient for well-being from T1 to T2 is quite small (boys, 0.44; girls, 0.34), indicating that there were notable changes in adolescents’ well-being between the two measurement occasions. Both the stability of achievement and the changes in the adolescents’ self-reported well-being are consistent with previous findings, as prior academic achievement is one of the strongest predictors of subsequent achievement (Khattab, 2015) and well-being generally declines from early through mid and late adolescence (Herke et al., 2019).

In the next step the cross-lagged associations were investigated, which were estimated while controlling for the correlations within time points between the constructs and their variance across time (the autoregressive associations). First, whether adolescents’ academic achievements by the end of compulsory school were related to changes in their subjective well-being in upper secondary school (from T1 to T2) was examined. As can be seen in Fig. 1, for boys, the cross-path coefficient from achievement at T1 to well-being at T2 is not statistically significant (−0.06, *p* = 0.223). However, the cross-path from the girls’ academic achievements at T1 to their well-being at T2 has a statistically significant, negative coefficient (−0.12), implying that higher grade points at graduation in 9^th^ grade were associated with lower well-being at T2.

Considering the other direction of the relationship, the cross-path from boys’ well-being at T1 to their achievement at T2 is not statistically significant (−0.04, *p* = 0.254). For girls, on the other hand, the corresponding association has a statistically significant, positive coefficient (0.11, *p* = 0.003), suggesting that higher well-being at T1 was associated with higher school grades at T2. The academic achievements influenced changes in the well-being of adolescent girls to a similar degree as well-being influenced changes in their achievements (−0.12 vs. 0.11), although in opposite directions (negative vs. positive). Taken together, these results lend some support for bidirectional relations between the constructs even across such a long time span as 2 years, but only for girls. These results also show that the direction of associations is not entirely straightforward when gender is also considered.

Sensitivity tests were performed in order to test the robustness of the results. It is possible that participants with very high grades and/or well-being at T1 had no room for improvement, and thus were more likely to have experienced declines compared to those who had low grades and/or well-being at T1. In order to take into account the potential of such ceiling effects, analyses were conducted in which participants with very high well-being and high grades at T1 were excluded, to examine whether the results differed. First, participants with an overall grade sum (academic achievement at T1) equal to and greater than 300 were excluded (*n* = 65) and the bidirectional model was re-estimated. Recall that this variable (unstandardized) ranged from 0–320 in increments of 5. In a second step the participants with the highest possible score on the converted well-being variable at T1 were excluded (*n* = 73). Lastly the model was re-estimated with both these groups of participants excluded. The cross-path coefficients increased in size but only marginally, by at most 0.05 standard deviations compared to the model presented in Fig. 1 (see appendix for details). In addition, a model that included the 84 participants with missing information on well-being or grades at T2 was estimated, with the FIML method to account for these missing data. This only reduced the size of the cross-path from the girls’ academic achievements at T1 to their well-being at T2, by 0.02 standard deviations compared to the model presented in Fig. 1. Overall, these procedures rendered minor changes in effect sizes but not to such a degree as to alter conclusions.

## Discussion

A common conception in various educational discourses is that promoting adolescents’ well-being will enhance their achievements at school, which in turn will reinforce their well-being (e.g., Kolouh-Söderlund et al. 2019). An assumption underlying this idea is that there is a bidirectional relationship between educational outcomes and well-being. However, empirical evidence for this so-called “virtuous circle” is limited. The overall pattern in previous research indicates a weak correlation (Bücker et al., 2018) and a positive contribution of well-being to academic achievement (Amholt et al., 2020). The other part of the bidirectional process, the contribution of adolescents’ achievements to their well-being, has been far less studied. Thus, the aim of this study was to investigate the directionality of associations between subjective well-being and academic achievement across two timepoints during the course of mid to late adolescence.

The results showed that the relationship between well-being and achievement was influenced by adolescent gender: support for a bidirectional relationship was found for girls, but not boys. Among girls, the results suggest a reciprocal relationship even across such a long time span as 2 years. Interestingly, the associations did not point in the same directions: higher achievements at T1 were indicative of lower well-being at T2, whilst higher well-being at T1 indicated higher achievements at T2. The latter association (from well-being to achievement) is consistent with previous findings that gender moderated the relationship between one component of subjective well-being and achievement. A previous study found that higher life satisfaction at one timepoint was associated with higher GPA and higher math test scores at a later timepoint, among girls but not boys (Heffner & Antaramian, 2016). The data explored in the present study indicate that for the participants, at least among girls, the association from achievement to well-being was approximately equally strong as the association from well-being to achievement, though in opposite directions (−0.12 vs. 0.11).

An important question to address is why higher academic achievement was indicative of lower subsequent well-being for the girls. There are at least two lines of research that provide possible explanations. First, research on academic efficiency has shown that interventions aimed at improving student performance can make school less joyful (Jürges & Schneider, 2010). In addition, there may be a trade-off between student well-being and performance (Heller-Sahlgren, 2018), because effective teaching and learning practices, and the efforts associated with achieving top results, does not generate happiness (positive affect). On the contrary, it may be rather tedious. Another possible explanation comes from research on school burnout (Walburg, 2014): achieving high grades in grade 9 may have provided a short-term boost in well-being, but maintaining a continuously high level of performance after the transition to upper secondary school may have led to exhaustion due to a high study workload (see e.g., Salmela-Aro & Upadyaya, 2014). These lines of research suggest that academic practices focused on high performance, and long term pressure to achieve, which generally impact girls more than boys (Giota & Gustafsson, 2017, Östberg et al., 2015), may impair rather than promote well-being. However, no prior empirical study has shown that high-performing girls develop lower subsequent subjective well-being specifically, than less high-performing girls. Thus, more longitudinal research is needed to further evaluate this finding.

Among boys, contrary to expectations, none of the cross-paths were statistically significant. Previous studies have shown that Swedish boys and girls tend to differ in their valuation of educational attainment. Girls have reported that their future depends on doing well in school, while boys have not perceived school grades to be so decisive for their future (Östberg et al., 2015). This could explain the nonsignificant links between the constructs among boys. This result for boys adds to the heterogeneity of prior research which has provided mixed evidence: while some studies support a positive association between well-being and achievement, other studies have not detected any significant association between these variables (Amholt et al., 2020).

When interpreting results of this study, one should keep in mind the measure of academic achievement that was used, i.e., school grades, and its role in the existing education system. In the Swedish system, the grades obtained in school-year 9 (the last year of compulsory school) dictate students’ eligibility for upper secondary school programs, and those obtained in upper secondary school dictate eligibility for university programs. Grades in Sweden are thus selection instruments for further studies in the education system. Since bidirectionality between well-being and achievement implies feedback loops, the results indicate that school grades can be regarded as feedback instruments in this bidirectional process. As mentioned, adolescent boys and girls tend to value school grades differently (Östberg et al., 2015), so the grades they achieve may have differing effects on their well-being, and possible differences in the associated feedback loops warrant attention. Other student characteristics, such as personality and educational aspirations will most likely also determine the association.

Another aspect to bear in mind concerns the time lag, or time interval, between measurement occasions, i.e., the observable points in the presumed feedback loop(s). For instance, the positive reciprocal relationship between life satisfaction and academic engagement found in previous studies are likely related to the short time interval between their measurement points, e.g., 2 months (Datu & King, 2018) and 5 months (Lewis et al., 2011). These studies capture associations within a single school year. In contrast, the interval between measurement occasions in the present study extended over two to three school years and the transition from compulsory to upper secondary school. Thus, the degree of validity of the notion that “high levels of life satisfaction exert a positive influence on academic outcomes, which in turn boost future life-satisfaction” (Ng. et al., 2015, p. 487) may depend, at least partly, on the time intervals. Studies with short lags (time intervals) generally detect stronger bidirectional effects than those with longer delays between measurement waves (cf. Steinmayr et al., 2016). Conversely, increasing the time between measurement occasions raises the likelihood of not capturing some of the critical points in such feedback loops. The long time span between measurements in the present study could also be a reason why the detected effects were small.

Some further limitations of the study should also be mentioned. Well-being and academic achievement at T2 were assessed around a year apart, which violates the assumption of synchronicity. This is a limitation which should be kept in mind when interpreting the results. Ideally, the study would have included three or more waves of data with both well-being and achievement measures to assess the longitudinal relationships more comprehensively. The variables included in the missing analysis indicated no missing bias, except with regard to family structure. Accordingly, all analyses were adjusted for family structure. However, it is not possible to rule out other types of missing bias, and to definitely conclude that the participants are representative of the total population of adolescents in Umeå. Thus, the generalizability of the findings to the target population and the Swedish population in general, and societies outside Sweden, requires further exploration.

The study also has a number of strengths and extends the current literature in several ways. One is the use of longitudinal data, which enabled to focus on changes over time rather than static relations. Another is that school grades were considered as both outcomes and predictors of adolescents’ subjective well-being. Thus, the study expands the “unidirectional research paradigm” to include bidirectional associations. By considering the moderating role of gender the findings reveal some of the complexities in the relationship between well-being and achievement during adolescence. To gain further insights, since neither boys nor girls are homogenous groups, future studies should differentiate not only between boys and girls but also between different types of students with different characteristics. Future studies and the research field as a whole could benefit by using harmonized measures of constructs. Using the KIDSCREEN-52 instrument to measure well-being for instance, as in the current study, is advantageous from both a comparability and replication perspective. In addition, the focus on phenomena in the Swedish educational system limits the generalizability of the study’s results, but also increases the contribution to the research field from an international perspective, as no prior study on the topic has been published with data from Sweden. Taken together, the current study is a novel contribution to the research on the links between subjective well-being and academic achievement.

## Conclusion

There is a lack of research on the longitudinal and reciprocal links between adolescents’ subjective well-being and academic achievements. To bridge this research gap, the current study investigated the bidirectional relationship between subjective well-being and school grades across two timepoints during mid- to late- adolescence. The findings suggest gender differences in the relationship as no associations were found among boys. The findings indicate a bidirectional relationship only for girls, were higher well-being by the end of compulsory school predicted higher subsequent achievements, and higher academic achievements by the end of compulsory school predicted lower well-being in upper secondary school. Thus, not only may the well-being of adolescent girls be important for their ability to perform at school, but their academic achievements may also inflict negatively on their well-being. This suggests that promoting well-being in schools is important, and school personnel should pay extra attention to high achieving adolescent girls, and their sense of well-being.

## Appendix A

**Table:**

	Model 1	Model 2	Model 3	Model 4	Model 5
Boys					
Academic achievement T1 → Academic achievement T2	0.72 (0.67, 0.08)	0.69 (0.63, 0.74)	0.73 (0.67, 0.78)	0.69 (0.63, 0.75)	0.76 (0.71, 0.80)
Subjective well-being T1 → Subjective well-being T2	0.44 (0.36, 0.52)	0.44 (0.36, 0.52)	0.36 (0.26, 0.46)	0.37 (0.27, 0.45)	0.44 (0.36, 05)
Academic achievement T1 → Subjective well-being T2	−0.06 (–0.15, 0.03)	−0.04 (−0.14, 0.05)	−0.06 (−0.16, 0.05)	−0.03 (−0.14, 0.08)	−0.04 (−0.13, 0.04)
Subjective well-being T1 → Academic achievement T2	−0.04 (−0.11, 0.03)	−0.05 (−0.12, 0.03)	0.05 (−0.02, 0.13)	0.06 (−0.02, 0.14)	−0.04 (−0.10, 0.03)
Girls					
Academic achievement T1 → Academic achievement T2	0.72 (0.67, 0.77)	0.70 (0.65, 0.76)	0.71 (0.65, 0.76)	0.69 (0.63, 0.75)	0.73 (0.68, 0.78)
Subjective well-being T1 → Subjective well-being T2	0.34 (0.25, 0.44)	0.32 (0.22, 0.42)	0.34 (0.24, 0.44)	0.32 (0.22, 0.43)	0.36 (0.27, 0.45)
Academic achievement T1 → Subjective well-being T2	−0.12 (−0.22, −0.02)	−0.15 (−0.26, −0.05)	−0.14 (−0.24, −0.04)	−0.17 (−0.28, −0.06)	−0.10 (−0.19, −0.01)
Subjective well-being T1 → Academic achievement T2	0.11 (0.04, 0.18)	0.09 (0.02, 0.17)	0.14 (0.07, 0.21)	0.12 (0.04, 0.20)	0.11 (0.04, 0.18)

All estimates are standardised. Confidence intervals are in parentheses.

Confidence intervals that do not include 0 indicate that estimates are statistically significant at the 5% level (*p* < 0.05).

Model 1 = Estimates from the main model presented in the article (Fig. 1).

Model 2 = Participants with an overall grade sum (academic achievement at T1) equal to and greater than 300 excluded (*n* = 65).

Model 3 = Participants with the highest possible score on the converted well-being variable at T1 excluded (*n* = 73).

Model 4 = Both the above excluded.

Model 5 = All the participants at T2 included (*n* = 807), i.e., also those with missing information on well-being (*n* = 4) and school grades (*n* = 81).
